# The effect of bromelain in periodontal surgery: a double-blind randomized placebo-controlled trial

**DOI:** 10.1186/s12903-023-02971-7

**Published:** 2023-05-13

**Authors:** Hossein Babazade, Arad Mirzaagha, Shokoofeh Konarizadeh

**Affiliations:** 1 Department of Gingival Surgery, Faculty of Dentistry, Hormozgan University of Medical Sciences, Bandar Abbas, Iran; 2 Student Research Committee, Faculty of Dentistry, Hormozgan University of Medical Sciences, Bandar Abbas, Iran

**Keywords:** Bromelain, Hemorrhage, Periodontal pocket

## Abstract

**Background:**

Periodontitis is a persistent inflammatory condition. Eliminating the infection and reducing its risk factors are the first steps in treating periodontitis. When the anti-infective therapy is complete, there may still be deep periodontal pockets and prolonged inflammation. Surgical pocket reduction or elimination is indicated under these circumstances. We aimed to evaluate the effect of bromelain on bleeding on probing (BOP), gingival index (GI), and plaque index (PI) after pocket elimination surgery.

**Methods:**

This double-blind randomized placebo-controlled trial included 28 candidates for pocket elimination surgery referred to the private office of a periodontist in Bandar Abbas, Iran, from April 18 to August 18, 2021. Patients’ general characteristics, such as age and sex, were recorded. Additionally, periodontal indices including BOP, PI, GI, and pocket probing depth (PPD) were evaluated in all subjects. All patients underwent pocket elimination surgery. Afterwards, they were randomized into two groups. The first group received 500 mg Anaheal (bromelain) capsules twice a day before meal for one week. The second group received placebo, prepared in similar shape and color by the same pharmaceutical company. BOP, PI, GI, and PPD were assessed four weeks after completion of the treatment course (five weeks after surgery).

**Results:**

Four weeks after intervention, BOP was significantly lower with Anaheal compared to placebo (0% vs. 35.7%, P = 0.014). However, there was no significant difference in GI between groups (P = 0.120). Mean PI was lower (17.71 ± 2.12 vs. 18.28 ± 2.49) and mean PPD higher (3.10 ± 0.71 vs. 2.64 ± 0.45) in the Anaheal group, but the differences did not reach statistically significant levels (P = 0.520 and P = 0.051, respectively).

**Conclusions:**

One-week treatment with Anaheal at a dose of 1 g/d after pocket elimination surgery resulted in significantly lower BOP compared to placebo.

**Trial registration:**

Iranian Registry of Clinical Trials (IRCT), IRCT20201106049289N1. Registered 06/04/2021. Registered prospectively, https://www.irct.ir/trial/52181.

## Introduction

Periodontitis is a persistent inflammatory condition that my cause tooth loss by destroying the periodontal attachment apparatus [1]. As the disease worsens, the attachment apparatus migrates to the apex, resulting in the formation of a periodontal pocket. It is generally agreed upon that decreasing probing pocket depth (PPD) via different treatment modalities may optimize the prognosis of the individual tooth [2]. The initial stages in treating periodontitis are eliminating the infection and reducing its risk factors. This is primarily accomplished through a non-surgical method that aims to lessen gingival inflammation and decrease probing depth [3]. Deep periodontal pockets and ongoing inflammation may still exist after the anti-infective phase is complete. The recommendation in these circumstances is surgical intervention since this result is inadequate for effective supportive care. In addition to debridement with a direct vision, pocket reduction/elimination techniques also seek to alter the soft tissue’s contours and, on occasion, their hard tissue borders in order to reduce PPD in a controlled manner [4].

Nonsteroidal anti-inflammatory drugs (NSAIDs) are routinely used in clinical practice to treat the pain, trismus, and face edema that dental surgical procedures typically cause in the postoperative period. However, NSAIDs have multiple adverse effects, including issues with the gastrointestinal system, the kidneys, and the blood, particularly in older individuals [5]. Bromelain is a proteolytic enzyme found in pineapple plants. From inhibiting platelet aggregation to having anti-inflammatory actions, it exhibits a variety of biological features. Bromelain promotes reabsorption of fluid into blood circulation, increases tissue permeability via fibrinolysis, decreases existing edema, and inhibits edema development [6, 7]. We aimed to compare the effect of bromelain with placebo on bleeding on probing (BOP), gingival index (GI), plaque index (PI), and pocket probing depth (PPD) after pocket elimination surgery.

## Methods

### Participants and study design

The study received approval from the Ethics Committee of Hormozgan University of Medical Sciences under the ethics code: IR.HUMS.REC.1399.562. The trial has also been registered prospectively at the Iranian Registry of Clinical Trials (IRCT), IRCT20201106049289N1, available at https://www.irct.ir/trial/52181.

This double-blind randomized placebo-controlled trial was performed on candidates for pocket elimination surgery referred to the private office of a periodontist in Bandar Abbas, Iran, from April 18 to August 18, 2021. The inclusion criteria were age 18–70 years, chronic periodontitis, completion of phase I periodontal therapy, and PPD ≥ 5 mm. The exclusion criteria were systemic diseases and smoking. Patients who did not attend the office for follow-up were also excluded from the study. Written informed consent was obtained from all the patients. The sample size was calculated as 14 patients in each group based on α = 0.05 and β = 0.1.

Patients’ general characteristics, such as age and sex, were recorded. Additionally, periodontal indices including BOP, PI, GI, and PPD were evaluated in all subjects. Then, all patients underwent pocket elimination surgery by a single periodontist. Afterwards, they were randomized into two groups by coin flip. The first group received 500 mg Anaheal capsules (Salamat Parmoon Amin Pharmaceutical Co., Iran) twice a day before meal for one week. The second group received placebo, prepared in similar shape and color by the same pharmaceutical company. Anaheal and placebo were placed in similar bottles (only labeled A and B) by an assistant who was uninvolved in the study. Therefore, the patients, the caregivers, and the outcome assessor were blinded to the received medications. BOP, PI, GI, and PPD were assessed four weeks after completion of the treatment course (five weeks after surgery) by the researcher (outcome assessor).

### Data analysis

The Statistical Package for the Social Sciences (SPSS) software (version 25.0, Armonk, NY: IBM Corp., USA) was used for statistical analysis. Descriptive statistics, including mean, standard deviation, frequency, and percentage were used to describe the variables. Based on the results of the Kolmogorov-Smirnov normality test, the independent t-test was used to compare continuous variables between groups. The Fisher’s exact test was used to compare categorical variables between groups. P-value ≤ 0.05 was considered statistically significant.

## Results

**Fig. 1:**
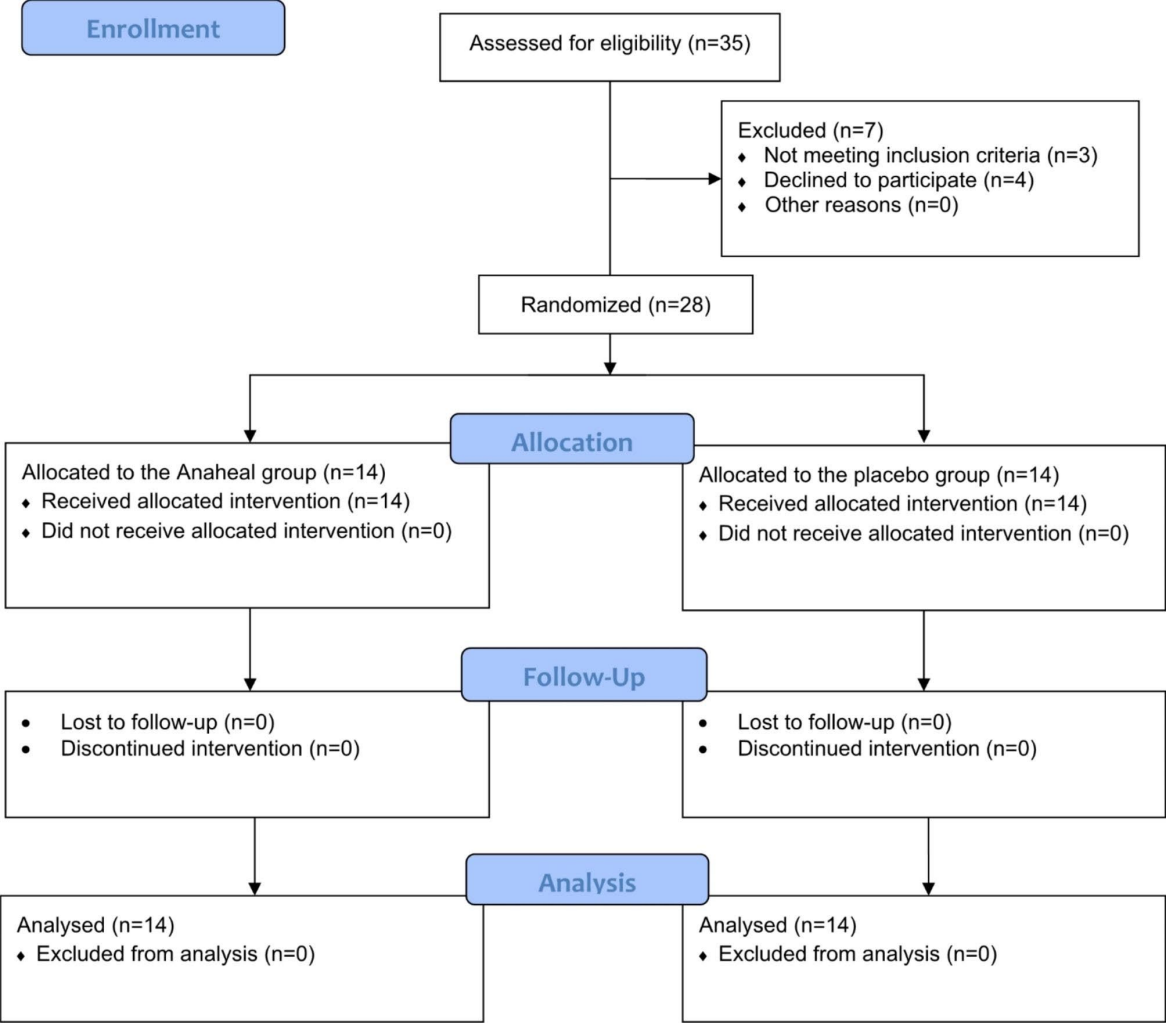
Details of patient enrollment, intervention allocation, and analysis

Initially, 35 patients were assessed for eligibility, of whom three did not meet the inclusion criteria, and four declined to participate in the study (Fig. 1). Table 1 shows the general and baseline characteristics of the participants.

**Table 1:** Comparison of general and baseline characteristics between groups

Variables	Anaheal (n = 14)	Placebo (n = 14)
Age (years), mean (SD)	44.78 (8.75)	43.95 (6.15)
Sex, N (%)		
Male	6 (42.9)	7 (50.0)
Female	8 (57.1)	7 (50.0)
BOP, N (%)	14 (100.0)	11 (78.6)
GI, N (%)		
1	1 (7.1)	4 (28.6)
2	13 (92.9)	10 (71.4)
PI (%), mean (SD)	18.00 (1.83)	19.07 (2.26)
PPD (mm), mean (SD)	5.03 (0.69)	5.07 (0.67)

Abbreviations: N, number; SD, standard deviation; BOP, bleeding on probing; GI, gingival index; PI, plaque index; PPD, pocket probing depth

Four weeks after intervention, BOP was significantly lower with Anaheal compared to placebo (P = 0.014). There was no significant difference in GI between groups (P = 0.120). Mean PI was lower and mean PPD higher in the Anaheal group; however, it did not reach statistically significant levels (P = 0.520 and P = 0.051, respectively) (Table 2).

**Table 2:** Periodontal indices four weeks after intervention

Variables	Anaheal (n = 14)	Placebo (n = 14)	P-value*
BOP, N (%)	0 (0.0)	5 (35.7)	0.014
GI, N (%)			
0	4 (28.6)	5 (35.7)	0.120
1	10 (71.4)	6 (42.9)	
2	0 (0.0)	3 (21.4)	
PI (%), mean (SD)	17.71 (2.12)	18.28 (2.49)	0.520
PPD (mm), mean (SD)	3.10 (0.71)	2.64 (0.45)	0.051

Abbreviations: N, number; SD, standard deviation; BOP, bleeding on probing; GI, gingival index; PI, plaque index; PPD, pocket probing depth

*Analyzed by the Fisher’s exact test

†Analyzed by the independent t-test

## Discussion

The results of the current study showed significantly lower BOP with Anaheal (bromelain) compared to placebo in patients who underwent pocket elimination surgery, four weeks after completion of the treatment course. Moreover, none of the patients in the Anaheal group had a GI score of 2. Also, Anaheal had a positive effect on PI, but without statistically significant difference with placebo.

When compared to or used in conjunction with other anti-inflammatory medications, bromelain has been utilized in dentistry for its anti-inflammatory properties, particularly following third molar extraction [8]. Among the anti-inflammatory medications used in periodontal surgery, corticosteroids are very commonly applied to prevent postoperative inflammation. Troiano et al. have reported no statistical difference in postoperative swelling, pain, and trismus after third molar surgery when dexamethasone was administered intraoral submucosal or extraoral intramuscular [9]. Nevertheless, corticosteroids’ mechanism of action is through reduction of fluid transudation, inhibition of vascular dilation, and decreasing the production of inflammatory mediators and inflammatory cells chemotaxis [9], which is different from bromelain and may be contraindicated in immunosuppressed patients. Moreover, bromelain has additional beneficial properties compared to corticosteroids’ anti-inflammatory features.

Experimental and clinical research has shown that bromelain offers a variety of benefits, such as speeding up the healing of wounds, debriding burns, and reducing pain. In addition to anti-inflammatory effects, it also possesses anti-edema, anti-coagulant, and platelet aggregation inhibitory features [10, 11]. The pH range of 4.5 to 9.5 is where bromelain is most active. It is absorbed via the intestines, where it stays physiologically active with a half-life of 6–9 h and a plasma concentration of 2.5 to 4 ng/ml. The main pharmacological actions of bromelain are influenced by its ability to break down proteins. Studies have demonstrated that oral bromelain has a dose-dependent effect on reducing the levels of bradykinin, plasmakinin, prostaglandin E2, and thromboxiane B2 in individuals with inflammation. In fact, bromelain specifically inhibits thromboxane and changes the thromboxane to prostacyclin ratio so that it favors anti-inflammatory prostacyclin [12, 13]. In addition to being non-toxic, bromelain is thought to be relatively safe and with no adverse effects [12, 14].

The efficacy of bromelain has previously been assessed following mandibular third molar surgery. Bhoobalakrishnan et al. showed that in the treatment of postoperative edema, discomfort, and trismus, bromelain was equivalent to diclofenac [15]. Moreover, when compared to diclofenac, bromelain demonstrated a non-significant, but equal, if not higher analgesic impact following third molar surgeries in two other studies [16, 17]. Besides, in a systematic review and meta-analysis by de Souza et al., bromelain proved successful in reducing the above-mentioned conditions [18].

Consistent with our findings, Banihashemrad et al. showed lower but non-significant GI after periodontal surgery of pocket elimination by Widman flap technique [19]; nevertheless, they used a different dose of bromelain in their study. On the other hand, contrary to our results, Zarandi et al. demonstrated significantly lower PPD, PI, and GI with bromelain than placebo, but comparable BOP. Nonetheless, the sample size was larger and patients received nonsurgical periodontal treatment instead of pocket elimination surgery in their study [20]. Furthermore, in a study on the effects of bromelain-containing toothpaste, it was shown that the GI and PI of the intervention group were considerably lower than those of the control group [21].

To our knowledge, this study is among the pioneers for the evaluation of bromelain effects after pocket elimination surgery, but it was not without limitations. We did not have an NSAID arm in our study to compare the effects of bromelain with. In addition, the sample size was relatively small which might account for the nonsignificant differences as P-values are largely dependent on sample size. Also, the small sample size can limit the generalizability of our findings. Further, we did not control the post-intervention results in terms of pre-intervention values.

## Conclusions

The results of the current study showed that treatment with Anaheal after pocket elimination surgery led to significantly lower BOP compared to placebo. It also had a positive effect on GI and PI. However, more research with a large sample size is necessary to corroborate these results. Also, adding an NSAID arm for comparison would be beneficial in future trials.

## Data Availability

The datasets used and/or analyzed during the current study are available from the corresponding author on reasonable request.
